# ‘When she rises, we all rise’: a crowdsourcing challenge to increase women’s participation in an infectious diseases research fellowship

**DOI:** 10.1186/s12879-020-05433-5

**Published:** 2020-09-29

**Authors:** Ewen Liu, Juliet Iwelunmor, Grace Gabagaya, Helen Anyasi, Alejandra Leyton, Karen A. Goraleski, Shufang Wei, Mariam Otmani del Barrio, Atinuke Olaleye, Pascal Launois, Joseph D. Tucker

**Affiliations:** 1 Wake Forest University School of Medicine, Winston Salem, USA; 2 Social Entrepreneurship to Spur Health (SESH), Guangzhou, China; 3 College of Public Health and Social Justice, St. Louis University, St. Louis, USA; 4 Makerere University- Johns Hopkins University Research Collaboration, Kampala, Uganda; 5 FHI360, Abuja, Nigeria; 6 Tulane University School of Public Health and Tropical Medicine, New Orleans, USA; 7 American Society of Tropical Medicine and Hygiene, Arlington, VA USA; 8 Special Programme for Research and Training in Tropical Diseases, TDR, Geneva, Switzerland; 9 Department of Obstetrics and Gynecology, Babcock University, Ilishan-Remo, Nigeria; 10 Faculty of Infectious and Tropical Diseases, London School of Hygiene and Tropical Medicine, London, UK; 11 Institute for Global Health and Infectious Diseases, University of North Carolina at Chapel Hill, Chapel Hill, NC USA; 12 University of North Carolina Project-China, Guangzhou, 510095 China

**Keywords:** Fellowship, Training, Challenge contest, WHO

## Abstract

**Background:**

Women are under-represented in many mid-career infectious diseases research fellowships, including a TDR fellowship for low- and middle-income country (LMIC) researchers. TDR solicited creative ideas as part of a challenge contest to increase the number of women fellowship applicants. The purpose of this study is to examine themes from submitted ideas and the impact of implementing the top three ideas on the number of women applicants.

**Methods:**

We solicited ideas for modifying the TDR fellowship using a crowdsourcing challenge. Then we used a mixed methods approach to evaluate texts submitted in response to the challenge. The qualitative analysis identified themes from eligible submissions. The quantitative analysis examined the mean score (1–10 scale) assigned to submitted ideas and also the number of eligible women applicants before (2014–7) and after (2018) implementing the top three ideas.

**Results:**

We received 311 ideas on improving women’s participation in this fellowship from 63 countries. Among all ideas, 282 (91%) were from women and 286 (92%) were from low- and middle-income countries (LMICs). Thirty-three (17%) ideas received an overall mean score of 7.0 or greater. The top three ideas included enhanced social media communication targeting women, improving career mentorship, and creating a nomination system to nudge women applicants. These ideas were implemented as part of the 2018 fellowship application cycle. The number of eligible women applicants increased from 11 in 2016 to 48 in 2018. The number of eligible men applicants increased from 55 in 2016 to 114 in 2018. Women represent 44% (8/18) of the 2018 cohort.

**Conclusion:**

This suggests that the challenge contest resulted in strong participation from women in LMICs. The three top ideas likely contributed to a greater number of women applicants to this mid-career fellowship. Further ways of enhancing women’s participation in global health training are needed.

## Background

Women comprise an estimated 70% of the world’s global health workforce and most frontline health services are provided by women [1]. However, women are conspicuously under-represented at leadership levels in global health organizations [1–3]. The Global Health 50/50 report highlighted that 72% of executive heads and board chairs of prominent global health organizations are men [4]. The large number of women at the lowest levels of global health organizations and the smaller number of women at the highest levels suggests that women do not advance up through global health careers similar to men. Instead of a glass ceiling, women face an inefficient and precarious pipeline all the way up to the top [1]. A lack of gender-responsive global health training opportunities thwarts women at many stages of their careers. Mid-career global training opportunities are often limited [2, 5, 6]. Recognizing the need to enhance gender equality in its research impact assessment, the UNICEF/UNDP/World Bank/WHO Special Programme for Research and Training in Tropical Diseases (TDR) identified that only one-third of its mid-career global clinical research fellows were women. The number of women applicants to the fellowship was similarly low (20%) [7].

Responding to the need for greater women’s participation in this mid-career fellowship, TDR organized a crowdsourcing challenge to identify creative solutions. Crowdsourcing has a group attempt to solve a problem and then implements solutions (Table 1) [8]. Challenge contests provide an opportunity to solicit feedback from the public about a problem [9]. The purpose of this contest was to identify feasible ideas that could be used to increase the number of women applicants to a TDR mid-career clinical research fellowship. Supported by the Bill & Melinda Gates Foundation, the fellowship is designed to provide individuals from low- and middle-income countries (LMICs) with a one-year opportunity to undertake mentored clinical infectious diseases research through placements in pharmaceutical companies, product development partnerships, and research institutions.

**Table 1:** Overview of crowdsourcing contest stages, structure, and function (adapted from TDR 2018)

*Contest Stage*	*Structure*	*Function*
**1. Organize** community steering committee	Diverse group of individuals, previous fellows and gender experts	Finalize call for ideas, prizes, and contest rules
**2. Engage** community to contribute	Social media	Clarify the contest, encourage ideas
**3. Evaluate** contributions	Steering committee and other judges evaluate based on criteria	Narrows the field of ideas, identifies excellent ideas
**4. Recognize** exceptional finalists	Certificate for excellent ideas, participation in WLGH Conference	Officially acknowledges and celebrates finalist ideas and those who submitted
**5. Share** selected ideas and implement	Incorporate best ideas into practice or evaluate impact	Use best ideas to improve health services or campaigns

Much of the literature on increasing women’s participation in global health leadership has narrowly focused on individual issues instead of addressing the larger social and institutional structures that privilege men [10]. Many studies on sex parity in global health have been organized by researchers in the global north, with limited input and direction from the global south [1, 5, 11, 12]. This challenge contest created an opportunity to hear the voice of women in the global south and others. The objective of this study is to examine the challenge contest, thematically analyze textual ideas, and examine the effect on TDR fellowship policies.

## Methods

### Challenge contest

The challenge contest was hosted by SESH (Social Entrepreneurship to Spur Health) and TDR and called the “Women Leaders in Global Health Challenge” to complement the Women Leaders in Global Health Conference organized by the London School of Hygiene and Tropical Medicine [13]. We followed the TDR “Crowdsourcing in Health and Health Research Practical Guide,” and used the following steps: organizing a multisectoral steering committee; engaging the community to contribute; evaluating ideas based on pre-specified criteria; recognizing exceptional finalists through commendation at the Women’s Leaders in Global Health Conference; and implementing selected ideas to adjust TDR fellowship policy [9].

The challenge steering committee organized the challenge contest. A multisectoral steering committee included key stakeholders from diverse geographic regions. These included women who previously received this TDR fellowship, gender research experts, and public health experts. Countries represented included China (1), Germany (1), India (1), Kenya (2), Malaysia (1), South Africa (2), Switzerland (1), Tanzania (1), Tunisia (1), United Kingdom (4), United States (7), and Zambia (1). The steering committee met via Zoom teleconference on a monthly basis to guide challenge contest design, promotion and evaluation.

An open call was disseminated that explained the purpose of the contest and solicited short descriptions of solutions to promote women’s participation in this TDR fellowship [14]. We included quotes and photographs from women who had joined the fellowship before in order to provide context. A short video was posted online to clarify contest rules. Confidentiality was maintained throughout the challenge. The contest was open to anyone from any location, but ideas from women in low- and middle-income countries were particularly encouraged. We used the TDR website, social media platforms (Twitter, Facebook, YouTube, and WeChat), email listservs and networks of collaborating organizations represented by the steering committee to disseminate the call for entries.

All submitted ideas were screened for eligibility and then judged. Each idea was screened for eligibility by at least two independent individuals (EL, SW). Eligible ideas were relevant to the topic, written in English, and under 500 words. Each eligible idea was then independently evaluated by three members of the steering committee. They rated ideas according to pre-specified criteria: capacity to increase the number of women who apply and receive TDR fellowships; feasibility; and innovation, defined as different from the current practice used in the fellowship. Ideas were scored on a single 1–10 scale. Steering committee members who had a conflict of interest recused themselves accordingly. Conflicts were defined as collaborating, co-authoring, helping, receiving or providing monetary or other support, or anything that could be perceived as a conflict of interest. Mean scores of the three scores were calculated for each idea.

A subset of individuals who submitted ideas were recognized by commendations (pre-specified as ideas with mean scores greater than 7.0) and selected finalists from this group were supported to join the Women Leaders in Global Health Conference. Finalists were selected by the steering committee based on the same criteria. Then, we created a working group composed of finalists, TDR representatives, and fellow alumni to refine ideas and present them at the 2018 Women Leaders in Global Health Conference in London. All of the finalist ideas were also presented to TDR for consideration and they ultimately selected three ideas to modify the mid-career fellowship.

### Quantitative and qualitative methods

Summary statistics described gender identity, nationality, WHO region, and mean scores associated with ideas. In addition, we undertook a textual analysis of the ideas using qualitative methods. First, we developed a thematic codebook to classify and analyze themes. Themes were organized using Excel (Office 365, Microsoft). Two independent individuals with training in qualitative methods examined the top 50 ideas in order to identify potential themes. Themes were discussed with two qualitative researchers in order to finalize the code book. All eligible submissions were coded by three trained individuals. Discrepancies were brought to two additional study team members for discussion and resolution. Higher order themes were then iteratively identified and further characterized by example quotations.

We collected information from TDR about the total number of eligible men and women applicants to the mid-career fellowship before (2014–7) and after (2019) implementing the top three ideas. SPSS (version 26.0, IBM SPSS Statistics) was used for quantitative analysis.

## Results

### Challenge contest

**Fig. 1:**
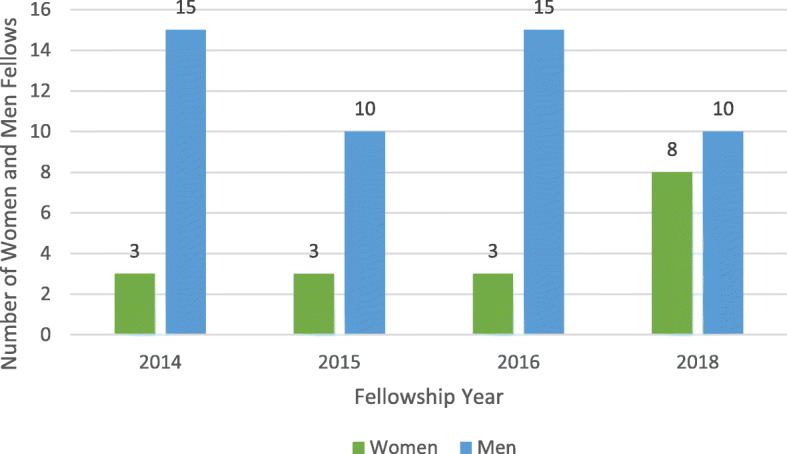
Total number of women and men fellows by year. The fellowship was altered between. 2016 and 2018 based on the outputs from the challenge contest

We received 311 ideas from 63 countries (Fig. 1). Two hundred and eighty-six ideas (92%) were from LMIC and the largest number of ideas were from Nigeria, Uganda, Egypt, Kenya, and India (Supplementary Figure 1). In terms of WHO regions, we received 146 ideas from Africa, 68 ideas from Eastern Mediterranean, 35 submissions from South-East Asia, 28 submissions from the Americas, 20 submissions from Western Pacific, and 14 submissions from Europe. Among all 311 ideas, 192 were eligible based on our pre-specified criterion. The 192 eligible ideas received an overall mean score of 5.63. Thirty-three (17%) of ideas received an overall mean score of 7.0 or greater (Supplemental Figure 2).

Two hundred and eighty-two ideas (91%) were from women. Among the 33 commended ideas, 29 (88%) were from women and 27 (82%) were from LMICs. There was a wide range of quality in ideas, including many exceptional ideas (Supplementary Figure 2). Among the 33 commended ideas, 29 (88%) were from women and 27 (82%) were from LMICs. Furthermore, five WHO regions were represented in the top 33 ideas, including nineteen from Africa, six from the Americas, five from South-East Asia, two from Western Pacific, and one from Europe.

### Qualitative analysis

We identified several themes from ideas to increase women’s participation. The themes that emerged from the analysis focused on improved career mentorship, targeted communications strategies, a nomination system within the fellowship, and gender responsiveness. Gender responsiveness can be defined as a program or policy that considers gender norms, roles, and inequality alongside actions to reduce their harmful effects [15].

### Improving career mentorship

Eighty ideas (42%) discussed increasing opportunities for career mentorship, including pastoral issues, professional development, and logistics (Supplementary Table 1). Women noted that there were relatively fewer role models and mentors to help guide them in global health research. Many ideas mentioned identifying women mentors to provide social and technical guidance during the fellowship application process. This mentorship could provide practical advice to interested individuals about balancing personal and professional obligations during the fellowship. Several ideas mentioned inviting previous fellowship alumni to serve as fellowship mentors. Some ideas suggested enhanced mentorship for potential fellowship applicants such as graduate students and early-stage investigators (e.g. postdoctoral fellows, junior faculty). This could help build a pipeline of interested women scientists aspiring to join the mid-career fellowship. Creating women mentor champions was another suggestion. One finalist described her own experience trying to advance her career in a culture that values caregiving more than career advancement among women. She proposed a pre-application mentorship program that “allows for mentors to share experiences, expertise, values, and skills and for mentees to share their questions, doubts and worries.”

In response to this suggestion, the TDR fellowship invited three fellowship alumnae to serve as champions for the fellowship. The purpose of the champion program was to answer any questions about the fellowship through email or WhatsApp. Fellowship champions offered support on topics such as ‘balancing family and work,’ as well as nuanced biographies describing both their research and family experiences during the fellowship. Men, women, and non-binary people could ask champions about the fellowship. The fellowship champion program was advertised through an independent website. Between 1 January – 7 March 2019, the three fellowship champions spoke with 61 potential applicants from 22 countries, including 37 men and 24 women.

### Targeted communication strategies

The need for more effective fellowship communications materials and dissemination was mentioned in 90 (47%) ideas. Although previous TDR fellowship communications disseminated the call for entries through email listservs, the addition of social media announcements could enhance dissemination. Many ideas elucidated the need to be conscious of differences in common social media platforms in LMIC settings. Ideas included announcing the call for applicants through social media; disseminating the call through established social networks (e.g., UN Women, Women in Global Health, Global Health 50/50); and including content which describes both professional and personal aspects of the fellowship. Other suggestions included enhancing fellowship communications materials to explicitly display the ways that the fellowship already supports women applicants who are more likely to have caregiving responsibilities. For example, in some cases TDR allows one home visit during the fellowship.

In response, TDR implemented these changes by creating a website that included links to previous women fellow profiles and explicit statements in support of gender equality and women’s participation in the fellowship. TDR also supported the dissemination of call for application announcements through Twitter, Facebook, and LinkedIn.

### Nomination system for applicants

Finally, one finalist recommended creating a nomination system to encourage women applicants. The nomination system was based on the premise that small nudges from peers, co-workers, or friends may increase confidence in applying for a fellowship. In response to this suggestion, the TDR fellowship implemented a nomination system where any individual could nominate their peers, colleagues, or friends. This form was also advertised through the newly implemented website. The nominator reviewed fellowship eligibility prior to submitting their nomination. Among those with eligible colleagues, an automatic email was sent stating that someone had nominated them to apply for this fellowship. For ineligible nominees, further clarification of the fellowship was provided. The nomination system was used 88 times, inviting 64 eligible potential applicants to apply. Thirty-four women and 30 men were nominated.

### Gender responsiveness

Many ideas suggested the need for the fellowship program to implement more gender-responsive policies. Specifically, 86 (45%) ideas discussed the need for changes in the present fellowship structure to recognize gender roles and inequalities often associated with women’s caregiving responsibilities. In reality, many women disproportionately bear the burden of caregiving, complicating considerations about leaving their families in their home country for one entire year. Proposed fellowship changes included providing additional social support such as additional funds for child support or assistance finding a position for spouses. Another common theme was the need for support for the fellow when returning to their home institution and family, as career-oriented women in LMICs more often face resentment by colleagues/supervisors due to harmful gender norms, roles, and relations, including unequal power relationships.

### Women fellowship applicants

These three changes to the fellowship occurred immediately prior to the 2018 cycle. In 2018, the overall number of eligible applicants and fellows substantially increased (Supplementary Figure 3). The number of eligible women applicants increased from 11 in 2016 to 48 in 2018, while the eligible men applicants increased from 55 in 2016 to 114 in 2018. The proportion of women applicants increased from 16.7% (11/66) in 2016 to 31% (48/155) in the 2018 call for applications. The number of women fellows also increased from three in 2016 to eight in 2018 (Fig. 1).

## Discussion

Women are under-represented in many global health training programs, including the TDR mid-career fellowship. We organized a crowdsourcing challenge to identify creative ideas to increase women’s participation in this mid-career fellowship. The challenge resulted in 311 ideas from 64 countries. The challenge solicited powerful voices from women in the global south, directly impacting how TDR organizes its mid-career fellowship. This project was unique because it focused on a critical mid-career period of global health training, used a crowdsourcing challenge to identify ideas, and worked in close partnership with a global institution responsible for creating a global health training program.

We found that this crowdsourcing challenge engaged many women. This is consistent with other crowdsourcing challenges that have engaged large numbers of women [16]. This also aligns with other literature on crowdsourcing challenges suggesting that contests can enhance inclusion of some groups that are often marginalized [9]. Strong engagement of women may have been related to the topic of increasing women’s participation, the prize of joining a conference on women leaders in global health, or strong engagement through gender-related social media networks. This suggests that women should be strongly engaged when re-imagining global health training opportunities that are more gender responsive.

Our crowdsourcing challenge also engaged a substantial number of people from the global south. Ninety-two percent of all ideas were from people in low- and middle-income countries. This contrasts with much of the engagement literature on internet-based challenges, which typically draw greater numbers of participants from high-income countries [17, 18]. Furthermore, this engagement may have been related to the greater relevance of the fellowship there (only LMIC residents are allowed to apply) and our a priori focus on soliciting ideas from LMIC settings.

Our experience suggests that crowdsourcing methods may be feasible for developing innovative ideas to change global health training. The contest generated hundreds of ideas to increase women’s participation in global health training, with many ideas focused on a single fellowship. This method contrasts conventional ways of both developing and improving training fellowships. Literature shows that the initial steps of forming training fellowships depend on committees, whose composition do not reflect sex parity [19]. Evaluating training fellowships typically involves surveys of previous fellows [20, 21]. While surveys can help to evaluate program activities, they will not capture perspectives of individuals who did not apply for the fellowship. Our experience suggests that challenges might complement existing structures for re-configuring institutional policies related to training programs.

Our study has several limitations. First, the challenge contest did not use in-person engagement, which has been previously found to facilitate generating higher-quality solutions [22]. The number of men who participated was small. While women continue to pursue efforts for equal rights and opportunities, men must also be engaged to transform the patriarchal gender norms which disadvantage women, let alone people with non-binary identities. Second, many ideas we received might be relevant to increasing the number of women applicants at other global health training programs (e.g., fellowships organized by the Wellcome Trust, Ford Foundation, Medical Research Council, and US National Institutes of Health). However, this project focused on solutions which could be feasibly implemented in the context of a specific TDR fellowship. Finally, we only have reported short-term results from our most recent call for applications. Longer term follow-up may be useful for ensuring that the number of women applicants and fellows can be sustained.

## Conclusions

Our project demonstrates that a crowdsourcing challenge can effectively solicit large numbers of creative ideas from women in LMICs about how to increase the number of women fellows in a mid-career fellowship. Exceptional ideas from the crowdsourcing challenge were effective in increasing the number of women in the mid-career fellowship. This crowdsourcing challenge contest has implications for developing global crowdsourcing contests and increasing the voice of women in global health training programs. The findings of this study would be relevant to designing community-engaged research and programs in LMIC settings. Further implementation research is needed.

## Supplementary information

**Additional file 1.**

## Data Availability

The datasets used and/or analyzed during the current study are available from the corresponding author on reasonable request.

## Abbreviations

LMIC: Low and middle-income country
SESH: Social entrepreneurship to spur health
TDR: UNICEF/UNDP/World Bank/WHO Special Programme for Research and Training in Tropical Diseases
WHO: World Health Organization
