# Supplementary material for: ‘When she rises, we all rise’: a crowdsourcing challenge to increase women’s participation in an infectious diseases research fellowship
Source: BMC Infect Dis. 2020 Sep 29;20:715. doi: 10.1186/s12879-020-05433-5 (PMC7526393; doi:10.1186/s12879-020-05433-5)
Supplement: Supplementary file 1 — Additional file 1. [file 12879_2020_5433_MOESM1_ESM.docx]

**Supplementary Figures and Tables**

Supplementary Figure 1. Number of ideas from the top ten countries which contributed to the WLGH contest.


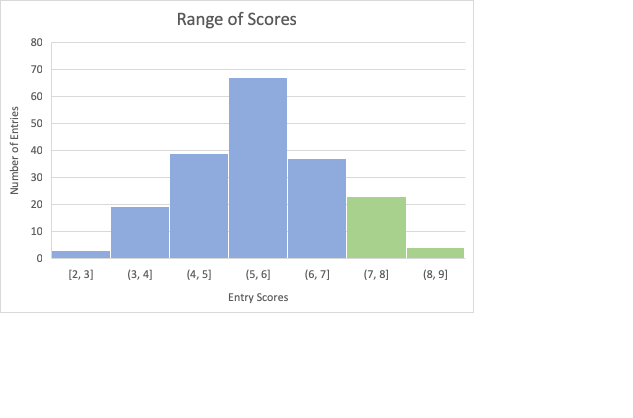


Supplementary Figure 2. Histogram showing range of scores of eligible ideas submitted to the WLGH contest (mean scores above 7.0 in green).

Supplementary Figure 3. Number of eligible applicants (women vs. men) for the fellowship from before the contest (2014-17) and after the contest (2018-19).

| Supplemental Table 1. Example quotations from eligible ideas. | |
| --- | --- |
| Theme | Quote |
| Mentorship | For a lot of women, seeing someone with a similar background in a role helps motivate them to apply. At the same time, having a support system, another female for example, helps as well. |
|  | Mentors can share their experiences of the fellowship (the research year) such as the schedule, the rewards it carries with it as well as the challenges. This is critical in the sense that it can allow one to work around that and be able figure out how they can say incorporate child care in their day’s schedule during the course of the fellowship and be able to strike a balance between career and family… A mentorship program can also provide an avenue for the mentees to share their doubts and worries with mentors who can relate to some of those concerns having walked the road and hence can offer insight on how they were able to deal with the respective concerns. |
|  | Past alumni who are interested can “pay it forward” by playing an active role in recruiting potential female participants by sharing information on their social media pages, doing sponsored promotional talks at popular women’s networking forums(SheLeadsAfrica, WIMBIZ), local scientific conferences and academic institutions in their home country. They can also offer to support and mentor potential participants on the application process. |
|  | Create a women-focused alumni network to share and to encourage women to learn from each other. Organize annual meetings and events. Give women experienced female mentors & coaches. |
|  | Having a mentor to guide you can help in encouraging the individuals in the programme during moments of uncertainty. This kind of mentorship will help guide them through not only the application procedure but also give them an idea on how to settle down after the fellowship programme. |
|  | I suggest that WHO/TDR could give consideration to creating regional platforms for dissemination of information on TDR fellowships. This platform would make use of past female grantees who are spread over the different geographical regions in LMIC to organize regional or country level orientation sessions for prospective female candidates. At these sessions, past female grantees in the respective regions could share their experiences of coping with the fellowships. This approach would not only to take information closure to the prospective candidates; but also provide the prospective candidates with source of help within their regions during the application process. |
| Communication  Communication | The first thing is:  I never hear about these things!  These days, most of my information comes through Social Media, Whatsapp Broadcasts, Women’s Forums and Conferences (in this order). Priority should be placed on information-sharing through:  Producing e-fliers packed with lots of attractive info-graphics that can be shared easily through social media. It should be downloadable @ the click of a button in PDF format. This is important as most people share these through e-mail, text messaging, Instagram or Facebook. |
|  | My attention was drawn to this challenge on the TDR clinical research and development fellowships from an automated email sent last month from the TDR website. Prior to this time, I had not heard about this fellowship. As a mid-career, female health professional myself working in a low to mid-income country, discussions with other colleagues revealed the same thing, none of them had heard of the fellowship. So, I think the first obstacle to surpass in getting more female scientists apply is lack of awareness. The fellowship should collaborate with women professional associations (e.g. Medical Women’s International Association) to disseminate the notice of the scholarship. Information from these Associations are usually sent as emails, which all respondents attested is a preferred means of communication and are treated as important. |
|  | As a new mother and at a not so young age, I completely understand the challenge of being at a place in your career where opportunities come knocking, some with great potential to advance my profession but my personal commitment to my infant, compelling me to ignore them…The global health symposium at Liverpool on its website displayed a section - child friendly HSR 2018 - which describes its own arrangements and also what is available locally in the city to support parents with children. This explicit display of support was welcoming for many parents like me looking to apply to Liverpool - I still need to figure it out but such acknowledgment lets me know that I am also wanted at the symposium. So for the TDR - bringing together local information and at the future workplace and making it available for applicants will be useful to let women know that TDR thinks of their families and their personal commitments and wants them to apply and support their fellowship. |
|  | The TDR clinical research and development fellowship organizers could participate in conferences hosted by low and middle-income countries (LMIC) research or academic institution, at which information about the programme can be disseminated… To increase awareness of the fellowship, in-bound and pre-roll video advertisement could be posted on the websites of LMIC research or academic institution, with activities relevant to TDR. These could be held on the sites for the duration of the fellowship announcement… Additionally, Social media advertisement (Facebook, Instagram, Twitter, YouTube etc.) could also be utilized; pictures and videos of the previous fellowship recipients could be posted. |
|  | Promotion: How and where the fellowship is advertised is vital. TDR would need to look at existing trends of sectors/institutions where female applicants tend to come from, as well as where they tend to hear about the fellowship so that publicity can be improvably targeted. If TDR is not already doing so, it will need to embed a question in the application pack about where applicants heard of the fellowship. |
|  | The fellowship should be advertised earlier in both timelines and professional trajectory. Applications should at least start being advertised on social media and clinical research institutions’ websites before November. Doctoral students should also be exposed to the fellowship’s existence before they complete their PhDs. The exposure and awareness that this fellowship exists, enables (post) doctoral students to have the full range of possible options to build their clinical research career. It enables the PhD students to build professional and personal trajectories whilst considering this opportunity along the way. You cannot make decisions about what you do not know exists. So, the more they know about this opportunity earlier in their career, the more capable they are of shaping decisions that incorporate the fellowship as part of their career. |
|  | Proposing a Crowdsourcing Women Networking & Mentorship Platform, the proposed innovation takes a page from the #meToo movement, where we saw women telling their stories, where they suffered sexual harassment at the hands of a male individual. The indictment of Harvey Weinstein was a significant example of what can be achieved with the movement. More survivors came forward nearly every day, many inspired and emboldened by those who have come before. |
| Making the fellowship gender-responsive | Society’s deep seeded assumptions about women in research inform expectations of teachers and peers. This perception can be directly and indirectly conveyed to girls, molding their own levels of interest and expectations. I am adamant that by fixing the systems that create this broken context, we can reverse the trends of women and build a more supporting ecosystem from early childhood through formal education and far into someone’s career… ‘when she rise, we all rise’ |
|  | Women often encounter gender-specific barriers in their professional lives and might worry about not being promoted upon return. A special funding for female alumni could be allocated for after the re-integration period (for example funding to hire two PhD students for their teams). |
|  | Support should be provided before and during the fellowship. Appointed TDR staff could help advise women in the pre-application, application and fellowship period on various (traditionally) women-specific issues, such as helping with visas for family members, childcare, home visits etc. |
|  | Traditional gender roles and expectations, particularly intensified in LMIC, mean that women as caregivers are less inclined than their male counterparts to accept a commitment that requires them to move away from home for 1year if they have a spouse and child(ren). A few measures which can be put in place and made evident at the application stage include:  • TDR positioning itself as willing to support women who cannot move with their spouses but can move with their children as it could be challenging for a spouse on a competitive career track to agree to pause that for 1year. This support can be in forms of working out a childcare and/or school arrangement/financing for children. If qualified women knew from the get go that there was an option which was not solely dependent on their spouse’s willingness/ability to move or having to leave their children behind, it could be an added advantage.  • TDR should consider allowing for breaks in-between fellowships, to be approved in special cases. The implication of this is that a fellowship aspirant knows that they could access the option of flying home once every quarter if it came to that, but at their own cost. |
|  | I must first state that I am a TDR fellow. I did my fellowship at GSK, Stockley Park, UK in 2010. I was actually the first fellow at that site. I first of all agree that it is very difficult for women to leave home for a whole year and more so when you have younger kids who definitely need a mother. I can cite myself as an example. I had to leave my 3 years - old son with his dad and a caregiver for almost 6 months. It was a very difficult decision I had to take at that time. I remember at my first outing with my team at GSK, someone asked me about my family and I cried out of guilt for leaving such a young child. I however applied for an academic visitor visa and this enabled me to bring my son, the care giver and my husband along later on during my fellowship. To enable women, especially those with family be able to apply for this fellowship to be able  to lead clinical trials and impact the research world… TDR should have a package that includes the possibility of bringing your  family along. |
|  | Mid-career women experience converging social, cultural, economic, and political factors that undermine their participation in the TDR Career Development Fellowship. A multi-dimensional approach that is oriented to address ecosystem bottlenecks that hinder women from applying, qualifying and participating for this fellowship is necessary to change the status quo. Increase the range of options available to women and trust them to make the decisions that best suit them. |
